# Cognitive Neural Mechanism of Sports Competition Pressure Source

**DOI:** 10.1515/tnsci-2019-0025

**Published:** 2019-04-25

**Authors:** Yucheng Zhou, Feifei Zhou

**Affiliations:** 1 Department of sports, Chongqing Jiaotong University, Chongqing 400074, China

**Keywords:** Cognitive neural mechanism, K-Means, Pressure resource, Competition stress

## Abstract

At sports events, the athletes by the pressure source is varied, based on the stress status of athletes, many athletes stress related experts at home and abroad to design questionnaire, questionnaire and sports events for athletes with often life process of in-depth and meticulous investigation, has formed the one whole set athletes pressure source of cognitive neuroscience assessment system, sports competition for athlete’s "escort". By participating in state general administration of sports scientific research project " management system of athlete competition pressure cognitive neuroscience" the development of using psychological pressure on athletes’ source data, the application of natural language processing and machine learning technology research these data, mainly using clustering algorithm and recommendation algorithm, thus forming pressure source research results are applied in sports competitions.

## 1 Introduction

Competition pressure is a special type of pressure which is mainly generated in the competition environment. Sports competition itself is very challenging, so it is inevitable for athletes to be in a state of stress for a long time[1]. General psychology and sport psychology research shows that competition pressure will give individual athletes and sports organizations to bring many negative effects, such as to cause anxiety, aggressive behavior, low satisfaction, and thus affect the competition results and health of body and mind. Poor coping skills will increase the muscle tension of the athlete, making the player’s attention and performance significantly lower[2]. Understand the players before the game after the source of stress and stress level is very necessary, and athletes only to fully understand and know their own stress factors, adjust their psychological state, will win the game in the arena [3,4].

In order to enable the athletes to play their competitive level reasonably, psychological professionals must make regular psychological counseling and psychological diagnosis for the athletes. Athletes psychological status, however, usually has the uncertainty and fuzziness, and most of the psychological service is to provide some simple corresponding method, unable to cope with cross complex actual condition, and with the increased number of athletes, psychological professionals can’t effectively consider the psychological status of each person [5]. How to effectively provide individualized service for individual athletes is a difficult problem. Traditional psychological counseling is usually conducted by psychological professionals, or some questionnaires are given to measure the psychological status of athletes. This article put forward the clustering algorithm and based on the content recommendation algorithm is applied to race, stress analysis and recommendations are given after clustering corresponding coping strategies, athlete pressure makes athlete facing pressure to improve psychological condition of the athletes is more compressive ability, bear setbacks difficulties psychological level be improved.

In this paper, by participating in state general administration of sports scientific research project “management system of athlete competition pressure cognitive neuroscience” the development of using psychological pressure on athletes’ source data, the application of natural language processing and machine learning technology research these data, mainly using clustering algorithm and recommendation algorithm, thus forming pressure source research results are applied in sports competitions.

## 2 Related theories and knowledge

### 2.1 Cognitive neuroscience

The study of cognitive neuroscience aims to elucidation the brain mechanism of cognitive activities, that is, how the human brain USES its components at all levels, including molecules, cells, brain tissue areas and the whole brain to realize various cognitive activities[6]. Some branches of traditional neuroscience absorbed the theory of cognitive science and neuroscience, new technology, gradually formed a cognitive neuropsychology, cognitive psychology, physiology, cognitive psychology, cognitive neuroscience and computational neuroscience and cognitive neuroscience of each branch. In the late 1980s, the research of cognitive neuroscience has made remarkable progress in a short period of time, which has a great impact on the theoretical construction and research of traditional cognitive psychology and developmental psychology. The study of cognitive development is no exception[7,8]. Since cognitive developmental psychology and developmental neuroscience are interested in many common problems, the derived developmental cognitive neuroscience is attracting more and more attention and has become one of the hottest cross-disciplinary research fields.

Competitive stressors refer to “environmental needs directly related to competition”. How to systematically summarize and sort out these needs has become the primary task of competitive stressors research. According to the specific sources of environmental needs, it can be divided into organizational pressure source and competitive pressure source. 14 elite athletes from different sports organizations were studied, and the results showed that the subjects faced a lot of competitive pressure sources and organizational pressure sources. Using code analysis, the researchers will be competitive pressure source divided into seven aspects: preparation, such as inadequate physical preparation, injury (such as the opponent’s flagrant foul), pressure (such as the result of the game directly decides the personnel selection), competitors (if you don’t understand the opponent), self (e.g., in the game to show the perfect shape) and events (e.g., performing complex technical movements), superstition, such as not take “lucky” equipment to match). And organizational pressure source is classified as four aspects: environmental issues, such as personnel selection unfair, the lack of financial support, training, facilities), personal problems, such as to his own expectations too high, the lack of goals and direction, and state for their high hopes), leading questions, such as tension coaches and athletes, coaches are not good at communication, coach too bossy, the coach’s professional skill not good), team issues (such as the lack of communication between teammates, his teammates adventurous, management did not fulfil his obligations, its role is not clear). The study also found that athletes reported more accurate amounts of organizational stressors and more diverse forms of performance than competitive stressors.

### 2.2 K-means clustering

Clustering is unsupervised learning that groups similar objects into a cluster. The clustering method can be applied to almost all objects. The more similar the objects in the cluster are, the better the clustering effect will be. K in the k-means algorithm represents the clustering of K clusters. Means to take the mean of the data values in each cluster as the centre of the cluster, or the centre of mass. That is to say, the centre of mass of each class is used to describe the cluster. The biggest difference between clustering and classification is that the goal of classification is known in advance, while clustering is different. Clustering does not know what the target variable is in advance, and categories are not defined in advance like classification. Therefore, clustering is sometimes called unsupervised learning. Cluster analysis tries to group similar objects into the same cluster and classify dissimilar objects into different clusters. Therefore, it is obviously necessary to find an appropriate similarity calculation method. There are many known similarity calculation methods, such as Euclidean distance, cosine distance, hamming distance and so on. In fact, we should select the appropriate similarity calculation method according to the specific application.

It is widely used as the k-means algorithm and is sometimes referred to as the Lloyd algorithm (especially in computer science) [9,10]. Given the initial k mean points $m_{1}^{\left(1\right)},\ldots ,m_{k}^{\left(1\right)}$false, the algorithm follows the following two steps:

**I) Assignment:**

Each observation was assigned to the cluster to minimize the sum of squares and (WCSS) in the group.

(1) $$S_{i}^{\left(t\right)}=\left\{x_{p}:{\left\|x_{p}:m_{i}^{\left(t\right)}\right\|}^{2}\leq {\left\|x_{p}:m_{j}^{\left(t\right)}\right\|}^{2}\forall j,1\leq j\leq k\right\}$$

Each *xp* false is assigned to a defined cluster *St* false, although in theory it may be allocated to two or more clusters.

**II) Update:**

For each cluster obtained in the previous step, the center of the observed value in the clustering is used as the new mean point.

(2) $$m_{i}^{\left(t+1\right)}=\frac{1}{\left|S_{i}^{\left(t\right)}\right|}\sum_{x_{j}\in S_{i}^{\left(t\right)}}x_{j}$$

Because the arithmetic average is the least square estimation, this step also reduces the value of the sum of squares and (WCSS) in the target function group. This algorithm converges when the distribution of observations is no longer changing. Since the two steps of alternating will reduce the value of the target function WCSS, and the allocation scheme is only limited, the algorithm is bound to converge to the optimal solution of some (local).

## 3 Application of K-means in stress analysis

With the rise of sports organizational behaviour, organizational stress has become a research hotspot. It breaks through the one-sidedness of previous researchers who only regard competitive pressure as an individual phenomenon and encourages researchers to explore more pressure sources and management strategies from the organizational level. In order to better promote the study of organizational stress, researchers need to learn from the theoretical model of job stress, especially the job demand-resources model (JDR). According to the JDR model, there are two different variables in any occupation. One is the work demand, that is, the pressure source, such as role conflict and work expectation; the other is the work resources, such as social support and organizational justice, which all include the four levels of physiology, psychology, organization and society. Generally speaking, work demands will consume employees’ physical and mental resources, leading to energy exhaustion and health problems, while work resources can help reduce work pressure and stimulate personal growth, learning and development. In sports organizations, athletes are also affected by job demands and job resources. Through the comprehensive analysis of job requirements and resource effect of consequence variables, such as the athlete burnout (athlete burnout), athletes, input (athlete engagement), will help to find out what kind of organization environment and atmosphere can cause harmful or beneficial result, thus provide beneficial enlightenment for the sports organization and management practice.

### 3.1 Data process

It consists of 23 assessment indicators, used for the frequency, intensity and duration of the movement of athletes asked a series of factors such as profiling. The questionnaire is translated from this questionnaire. By random questionnaire survey form, due to the cooperation with the school sports institute, the data source provided by the school sports institute, and the original data source is to the Wuhan sports college sports teams from 500 different levels of different hierarchical levels of the athletes have done a complete set of sports of psychological evaluation questionnaire data, 500 athletes of different projects, different grades and different ages filled in the questionnaire. A total of eight questionnaires, 22 kinds of attributes, and the questionnaire is divided into five grades, the psychological characteristics of general use: do not conform to the part, not completely conform to, in line with, most accord with, fully comply with the rank; General use of behavioral action: never, occasionally, sometimes, often, and always, the two are not fundamentally different. After obtaining the data, the questionnaire is quantified using 1 to 5 points.

I) Stress source questionnaire (# 11, 12, 16, 18, 20, 23); Logistics and Operations, (# 3, 5 6, 7, 14, 17, 19, 21, 22); Team of athletes (# 1, 8, 10, 13), mainly from team teammates, the relationship between team as the atmosphere of whole on its positive or negative factors; They are shown in table 1:

**Table 1:** Competitive pressure Source Questionnaire

1	Responsibility for a sports team
2	The relationship with the coach
3	The rules and regulations of sports teams
4	The character and attitude of the coach
5	Accommodation in training and competition
6	The attitude of the teammates
7	Age
8	The relationship between teammates
9	Selection of Eligibility for entry
10	Training arrangements
11	Organization of events
12	Injury

II) ** Mental toughness questionnaire.**

Confidence dimension: (# 13, 5, 11, 6, 14, l); Firm dimension, (# 3, 12, 8, 10); Control dimensions, (# 2, 4, 9, 7). They are shown in table 2:

**Table 2:** Mental Toughness Questionnaire

1	Once I become calm, I can recover immediately.
2	I’m afraid I’m going to give a bad performance.
3	I am committed to fulfilling my personal obligations.
4	I am haunted by my own suspicions.
5	I have no doubt about my ability.
6	I can also perform well when I am under pressure.
7	When things are not what I expected, I get angry and frustrated.
8	I choose to give up in the dilemma.
9	I will become anxious because things can’t be expected or controlled.
10	I am easily distracted and unable to concentrate.
11	I have a certain trait to differentiate myself from other competitors.
12	I will set myself a challenging goal.
13	I look at potential threats as a good opportunity.
14	Under pressure, I can make decisions with confidence and commitment.

III) Feelings questionnaire

Confidence dimensions (#1, 5, 9, and 13); Vitality dimension, (#2, 6, 10, 14); Dedication dimension, (#3, 7, 11, 15); The enthusiasm dimension, (#4, 8, 12, 16). They are shown in table 3:

**Table 3:** Athlete Input Questionnaire

1	I am confident that I can achieve my personal goals in sports.
2	I have a passion for training games.
3	I am committed to achieving my goals in sports.
4	I am very excited about my sports project.
5	I think I can achieve success in sports.
6	I feel energetic in the training competition.
7	I have the determination to achieve my goal of sports.
8	I’m passionate about my sports.
9	I believe I have the skills to succeed in sports.
10	I feel alive in the training competition.
11	I throw myself into my sports program.
12	I enjoy my sport very much.
13	I have great confidence in my ability.
14	I keep a high level of vigilance in the training competition.
15	I hope to achieve my goal by working hard.
16	I found a lot of fun in my sports program.

### 3.2 K-Means process

I) ** Data for clustering**

In chapter 3, the questionnaire is divided into: competition pressure source text, psychological resilience questionnaire, and exercise feeling questionnaire. Among them, the questionnaire is the most important in the competition pressure source. In addition, each questionnaire is divided into several sub-dimensions, and the detailed questionnaire and the subdivision dimensions are shown in table 4:

**Table 4:** Sports competition pressure Data dimension detailed description table

Datatype	Description	Datatype	Description
Varchar	Data number	Integer	Team
{0, 1}	Gender	Integer	Coach
Integer	toughness (confidence)	Integer	Selection
Integer	toughness (firmness)	Integer	Burnout (exhaustion)
Integer	input (confidence)	Integer	sense of achievement
Integer	input (Vigor)	Integer	Negative evaluation
Integer	input (devotion)	Integer	(emotional support)
Integer	input (enthusiasm)	Integer	(self-esteem support)
Integer	relationship intimacy	Integer	(information support)
Integer	Relationship commitment	Integer	(specific support)

After the evaluation of nearly 500 athletes, the comprehensive data are analyzed and the relevant clustering information is obtained. Due to the original questionnaire evaluation ranks generally divided into five segments (fully meet, compared with, general, more do not conform to, completely is not in conformity with the), so, get data raw score, score for each question 5 points, cumulative scores for the current breakdown dimension scores.

(3) $$X^{*}=\frac{X-min}{max-min}$$

According to the improved algorithm, after the data is cleaned and transformed, the clustering analysis should be carried out. Firstly, the contour coefficient should be used to calculate the approximate number of clusters K, figure 4. 3 represent the value curve of the contour coefficient (re) between 2 and 100. By figure 4 _3, the clustering number when 2 profile coefficient, maximum value indicating the clustering number when 2 effect is good, but this is not the final result, we can start with hierarchical clustering algorithm for clustering, and based on the improved algorithm, and calculate the next K-means the initial clustering center, convenient for subsequent calculations [11,12].

After calculation, the initial clustering center can be obtained as shown in table 5:

**Table 5:** Final cluster centers

Number	Final cluster center	Sum
1	(0.74 0.13 0.30 0.68 0.80 0.22 0.91 0.60 0.20 0.38 0.32 0.32	259
	0.30 0.09 0.36 0.32 0.22 0.72 0.71 0.73 0.34)	
2	(0.60 0.11 0.20 0.31 0.57 0.16 0.69 0.41 0.13 0.48 0.44 0.46	210
	0.11 0.49 0.48 0.42 0.47 0.47 0.52 0.24)	
3	(0.43 0.87 0.25 0.75 0.67 0.78 0.43 0.76 0.80 0.70 0.09 0.14	5
	0.44 0.88 0.42 0.43 0.62 0.66 0.66 0.57 0.82)

II) ** Result analysis**

Improved hierarchical K - Means algorithm to cluster the data sets, the source of competition pressure, social support, the athlete burnout 22 dimensions such as clustering, get the final clustering center as shown in table 5.

**Figure1:**
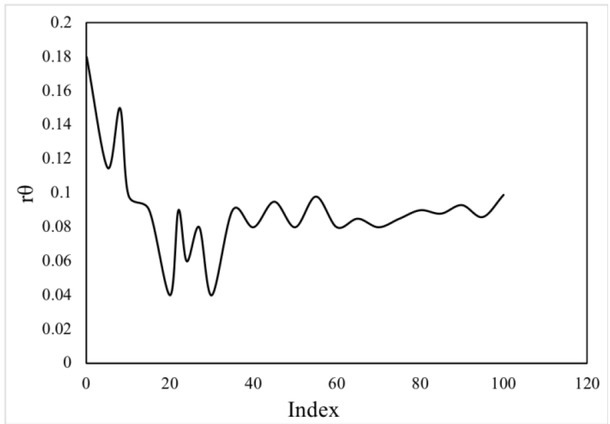
Silhouette Coefficient

**Figure 2:**
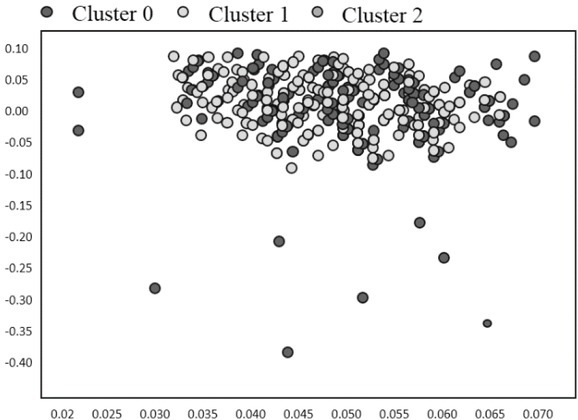
The pressure distribution of layered algorithm in sports competition

In figure 1 and figure 2 it can be seen that the first class occupies the most (blue line);

Category 2 second (green line), the third class at least (red line) type 1 and type 2 is bigger, the overall similarity part attribute is different, and the third class accounted for less than the total number, but have obvious difference with the class 1, 2, means that this part of the athletes psychological level is poorer (overall score is low, the pressure source does not conform to, less pressure).

## 3 Conclusions

Group 1 athletes, in general is good in toughness, but control is good in toughness, among them, the sense of control score is low, this is because the project is a reverse score, score is low, explain athlete mindset is good, the questionnaire shows that control aspect mainly refers to the athletes to their movement status, mindset rated items; Group 2 athletes compared with 1 class athletes, in terms of strength and commitment, relationship with the coach, compared with the first class, the difference is not big, are slightly lower than 1 class athletes score, belongs to medium level; Group 3 athletes with the former two analogy, in tenacity, lowest score, among them, the tenacity of the control points, this is the reverse calculation of breakdown, the project is mainly said the player he was depressed, have no confidence to oneself, thus influence sports, so need to strengthen, investment scale, the highest class 3 athletes enthusiasm, can maintain a good degree of enthusiasm [6]; In respect of competition pressure source, can see clearly that class 3 athlete’s greatest pressure source in the selection, followed by the competition pressure goals: finally, in the athlete’s burnout scale, negative evaluation scores highest motion, this shows that the third class athletes is easy to produce resistance to movement of sports question, lack of concentration, and then produce resistance.

The next step will be to integrate the results from neuropsychological, brain injury and functional imaging studies with the help of decision-making evaluation options, implementation of selection actions, and three-stage models of decision-making outcomes and learning processes in decision-making. Looking forward to is the future research direction
